# Endoscopic ultrasound-guided placement of lumen-apposing metal stent for transgastric drainage of loculated malignant ascites

**DOI:** 10.1177/26317745241289238

**Published:** 2024-10-14

**Authors:** Jacqueline Reuangrith, Stephanie A. Scott, Ali Kohansal

**Affiliations:** Division of Gastroenterology, Department of Medicine, Dalhousie University, 6299 South St, Halifax, NS B3H 4R2, Nova Scotia; Division of Gynaecology Oncology, Department of Medicine, Dalhousie University, Halifax, Nova Scotia; Division of Gastroenterology, Department of Medicine, Dalhousie University, Halifax, Nova Scotia

**Keywords:** ascites, drainage, endosonography, malignancy, stent

## Abstract

Endoscopic ultrasound-guided drainage of loculated malignancy-related ascites has been reported in limited case series with success in achieving symptomatic relief. In this case report, we detail the successful drainage of a loculated paragastric ascites with insertion of a lumen-apposing metal stent (LAMS) in a patient diagnosed with metastatic ovarian cancer.

## Plain language summary

Draining fluid buildup using a specialized stent: a case study in cancer treatment

This article discusses a specific procedure used to drain fluid buildup caused by cancer. The procedure involves using a special type of stent placed with the help of an ultrasound device. The case study shows how this method successfully relieved symptoms in a patient with advanced ovarian cancer.

## Introduction

Malignancy-related ascites can manifest in various cancers, including ovarian, colon, lung, breast, pancreatic and liver cancers. Its development often signifies poor prognosis and leads to substantial morbidity and impairment of a patient’s quality of life. Management of malignant ascites can be particularly challenging when it adopts a loculated state that is not amenable to percutaneous drainage. Recent case studies have introduced endoscopic ultrasound (EUS)-guided drainage with placement of plastic stents or fully covered self-expanding metal stents (FCSEMSs) as a promising treatment option.^1,2^

In line with these developments, we present a case wherein a LAMS was employed to successfully drain a loculated paragastric malignant ascites in a patient with ovarian cancer. This study adheres to the CARE guidelines, and patient consent for publication was obtained.

## Case report

**Figure 1.**
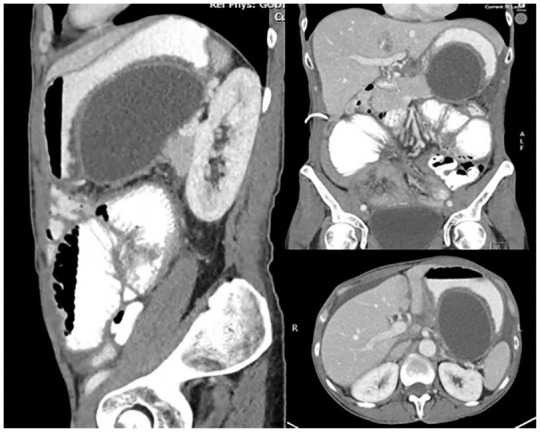
Sagittal, coronal and axial CT images of the large loculated malignant ascites at the posterior aspect of the stomach.

Our 60-year-old female patient was diagnosed with stage IV ovarian clear cell carcinoma with metastases to the liver and the peritoneum. Peritoneal fluid analysis revealed malignant cells consistent with metastatic adenocarcinoma. She had exudative non-neutrophilic ascites with a serum-ascites albumin ratio (SAAG) of less than 11 g/L, LDH of 2385 U/L and negative culture. She underwent a total abdominal hysterectomy, bilateral salpingo-oophorectomy and omentectomy with residual disease remaining. Despite several lines of systemic chemotherapy, her cancer progressed. Over 1 year after her surgery, she began experiencing a decline in her oral intake tolerance, accompanied by nausea and abdominal distension. As shown in Figure 1, she was found to have recurrent free fluid ascites and a new large loculated ascites situated at the posterior aspect of the stomach, measuring 8.8 × 5.4 × 14.4 cm. We determined that the loculated ascites was attributable to malignancy-related ascites, given the patient’s known peritoneal metastatic disease and the absence of alternative explanations. She had no history of acute or chronic pancreatitis, which makes pseudocyst formation unlikely, and her previous CT scans had not shown any pancreatic lesions suggestive of cystic neoplasms. Therefore, repeat cytology of the loculated ascites was considered unnecessary.

Due to its location, this collection was inaccessible for drainage through intervention radiology. In an attempt to alleviate her symptoms, an indwelling pigtail catheter was inserted to facilitate drainage of the peritoneal free fluid. The fluid analysis revealed non-neutrophilic ascites. This provided some relief, but she was readmitted a few weeks later with severe obstructive symptoms. Repeat imaging revealed a marked increase of the paragastric loculated ascites to 8.2 × 8.4 × 16.6 cm and proximal small bowel dilatation of up to 4.6 cm with an abrupt transition point in the left lower quadrant. She underwent EUS drainage with fine-needle aspiration on two occasions. Each time, there was removal of close to half a litre of fluid. Unfortunately, the fluid quickly reaccumulated. Following consultation with her endoscopist, a decision was made to pursue a more definitive treatment option with transmural drainage and stenting. The patient provided informed consent after a thorough discussion about the experimental character of the procedure and the potential risks.

**Figure 2.**
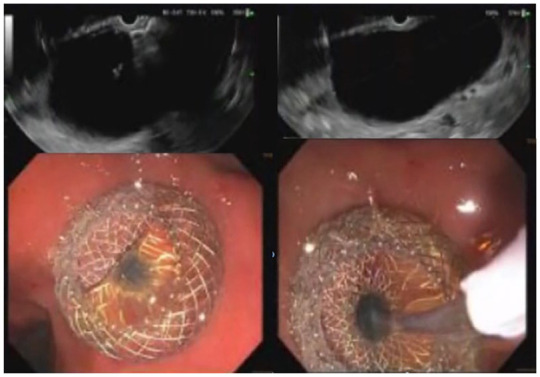
Transmural EUS-guided LAMS placement in the paragastric loculated malignant ascites. EUS, endoscopic ultrasound.

**Figure 3.**
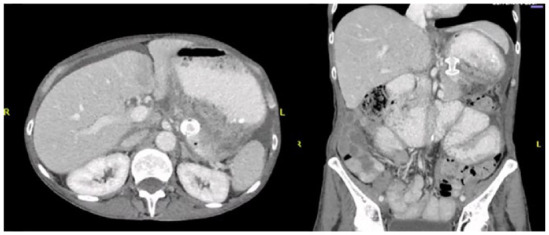
Axial and coronal abdominal CT images 1 week after EUS-guided transgastric placement of LAMS. EUS, endoscopic ultrasound.

Using a therapeutic echoendoscope, the walled-off ascites collection measuring 12 cm was identified, punctured with a 19-gauge needle and fluid samples were sent for cultures. As seen in Figure 2, a guidewire was then inserted, followed by the placement of a 10 × 10 mm Hot Axios Lumen-apposing fully covered self-expanding metal stent (LAMS). The procedure resulted in immediate drainage of clear ascites, and no immediate complications were observed. Prior to the procedure, she received an initial dose of intravenous ciprofloxacin, which was subsequently maintained. However, the ascites culture indicated light growth of *Klebsiella pneumoniae, Streptococcus gordonii*, and *Streptococcus salivarius*. Following consultation with infectious disease, it was felt that this was likely a contamination given the presence of less than three colonies as well as the absence of intra-abdominal infection symptoms. The antibiotic regimen was changed to amoxicillin-clavulanate for the remaining duration of the 7-day treatment course. As seen in Figure 3, CT scan conducted 1 week later showed a notable reduction in the size of the collection, diminishing from 8.2 × 7.6 × 8.2 cm to 7.6 × 2.7 × 2.9 cm after the transgastric stent placement.

Her recovery was uneventful, and she was able to progress to a solid food diet prior to her discharge. During her 1 -month follow-up appointment, she reported no signs of recurring obstructive symptoms and continued to tolerate oral intake quite well. There were no complaints of nausea, significant abdominal pain, or fever.

**Figure 4.**
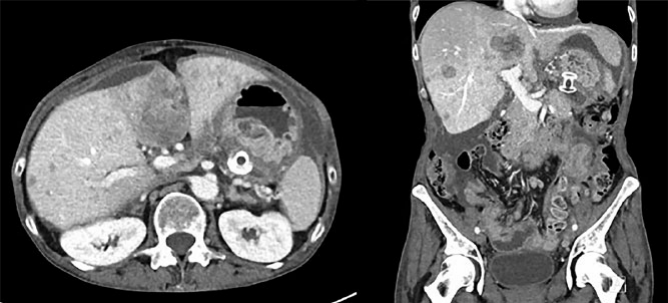
Axial and coronal abdominal CT images 3 months after EUS-guided transgastric placement of LAMS. ESU, endoscopic ultrasound.

Two months later, she was diagnosed with left leg deep vein thrombosis and extensive bilateral pulmonary emboli. Three months post the stent insertion, she was readmitted due to a recurrence of symptoms including nausea, vomiting, abdominal discomfort and difficulty tolerating oral intake. A follow-up CT scan, as seen in Figure 4, indicated progression of metastatic disease in her liver, spleen, bones and lymph nodes, without signs of bowel obstruction or significant recurrence of ascites. The gastric stent remained in a good position. It was believed that her symptoms stemmed from the extensive hepatic tumour burden and involvement of the peritoneum and mesentery, possibly affecting gastrointestinal motility. As a result, she was referred to palliative care for symptom management and passed away 4 months after her stent placement.

## Discussion

This is only the second reported case in the literature demonstrating the effective and safe use of LAMS for long-term palliative management of loculated malignant ascites. Malignant ascites accounts for about 10% of all cases of ascites and can be caused by several mechanisms such as peritoneal carcinomatosis, portal hypertension by liver metastasis, chylous ascites due to lymphoma, or occlusion of hepatic veins.^3^ In non-loculated ascites, serial paracentesis or the insertion of a percutaneous drainage catheter are frequently employed techniques for symptom relief. However, ascites can adopt a loculated state when it becomes confined by adhesions, malignancy, or infection. In these cases, the fluid becomes encapsulated, forming a distinct collection, that has the potential to exert a localized mass effect.^4^ Accessing loculated ascites for drainage can pose a challenge depending on its location. Successful cases have been documented in case reports such as one detailing the EUS-guide drainage in patients experiencing severe oesophageal and gastric obstruction caused by loculated malignant ascites. In these cases, authors used double plastic pigtail stents.^1^ Additionally, three other cases have been documented where malignant ascites were effectively drained using EUS-guided placement of FCSEMS.^2^

In recent years, EUS-guided drainage has become the mainstay of management for pancreatic fluid collections (PFC), with high technical and clinical success rates. Moreover, it is regarded as a safe procedure, with adverse events rate of around 5% in some studies.^5^ Lumen-apposing metal stents (LAMS) have demonstrated lower stent migration rates with similar procedure time, complication rates, technical and clinical success compared to tubular metal stents.^6^ In patients with malignant loculated ascites, there is limited literature comparing LAMS, self-expandable metal stent (SEMS) and plastic stent (PS), as only a few case reports exist. One of the main advantages of LAMS is the larger diameter provides a larger drainage lumen which is associated with a lower risk of stent occlusion and need to place multiple plastic stents. Our choice of stent was based on the availability at our centre, the size of the collection and high risk of re-accumulation. Larger diameter sizes are typically used for larger collections or those which contain thicker, necrotic debris.

Furthermore, higher rates of stent occlusion have been reported with PS and SEM stents compared to LAMS in patients with PFC.^7^ Stent occlusion due to gastrointestinal content or debris can lead to secondary infections. Using PS within a LAMS has been proposed as a strategy to reduce the risk of infection for PFCs. Authors of a prior case series incorporated several plastic pigtail stents inside an FCSEMS for loculated ascites, creating a barrier against solid particles.^2^ While this approach may also be considered, further research is needed to establish its effectiveness.

Infectious peritonitis is another potential complication that arises from the creation of a connection between the stomach and the peritoneum. To minimize this risk in patients undergoing transmural PFC drainage, antibiotic prophylaxis with broad spectrum coverage is given prior to the procedure and is typically continued for 3 days post-procedure.^8^ In this case, the patient received a longer course of antibiotics lasting 7 days.

Past studies on patients with PFC have indicated that LAMS was associated with a higher risk of perforation and delayed bleeding when compared to PS.^9^ Typically, cross-sectional imaging is performed after stent insertion to monitor the resolution of the collection, and LAMS are usually removed within 3–5 weeks to prevent impingement on adjacent intra-abdominal vascular structures and to minimize the risk of bleeding. It is unclear if the same risks apply to cases of loculated malignant ascites, as these collections are likely to be less inflammatory and contain fewer exposed vessels compared to a walled-off necrosis of the pancreas. For our patient, considering the palliative nature of the intervention, the potential for ascites re-accumulation, and the goal of minimizing endoscopic procedures, a joint decision was made to adopt a more conservative approach. Despite the stent remaining in place for over 6 months, the patient did not experience any stent-related complications.

## Conclusion

The positive outcome in our case highlights the potential effectiveness of the transmural drainage and stenting with LAMS, offering advantages such as improving visualization and reducing the need for multiple admissions and repeated procedures. This approach proves particularly valuable in cases of loculated ascites, where traditional methods may fall short. The current lack of guidelines poses a challenge to clinical decision-making, but we hope that further research and accumulation of similar cases will contribute to a deeper understanding of the role of EUS in managing loculated malignancy-related ascites. Further studies are required to define key aspects, including outcomes and complications with validated patient-related measures. This case report not only adds to the emerging body of literature on management of malignant ascites but also highlights the potential of LAMS as a viable and effective intervention.

## Declarations

**Ethics approval and consent to participate:** Informed consent was obtained from the patient for publication of this case report and any accompanying images. At our centre, ethics committee/IRB approval is not required for case reports.

**Consent for publication:** Informed consent was obtained from the patient for publication of this case report and any accompanying images.

**Availability of data and materials:** Not applicable.
